# Adaptation of an eHealth Intervention: iSupport for Carers of People with Rare Dementias

**DOI:** 10.3390/ijerph21010047

**Published:** 2023-12-28

**Authors:** Bethan Naunton Morgan, Gill Windle, Carolien Lamers, Emilie Brotherhood, Sebastian Crutch

**Affiliations:** 1 School of Psychology and Sports Science, Bangor University, Brigantia Building, Bangor LL57 2AS, UK; 2 School of Health Sciences, Bangor University, Fron Heluog Building, Bangor LL57 2EE, UK; 3 North Wales Clinical Psychology Programme, Bangor University, Brigantia Building, Bangor LL57 2AS, UK; 4 Dementia Research Centre, Queen Square Institute of Neurology, University College London (UCL), Queen Square, London WC1N 3BG, UK

**Keywords:** rare dementia, ehealth, internet, caregiver, carer, supportive intervention, adaptation, iSupport

## Abstract

‘iSupport’ is an online psychoeducation and skills development intervention created by the World Health Organisation to support people with dementia. This project adapted iSupport for carers of people with rare dementias (iSupport RDC), creating a new resource to support the health and wellbeing of this underserved population. The adaptation involved three phases: (1) Co-design methods to generate preliminary adaptations; (2) Analysis of phase one findings informing adaptations to iSupport to develop; iSupport RDC; (3) Post-adaptation survey to ascertain participant agreement with the adaptations in iSupport RDC. Fourteen participants contributed, resulting in 212 suggested adaptations, of which 94 (92%) were considered practical, generalisable, and aligned with iSupport principles. These adaptations encompassed content and design changes, including addressing the challenges of rare dementias (PCA, PPA, LBD, and FTD). iSupport RDC represents a significant adaptation of the WHO iSupport intervention. Its tailored nature acknowledges the unique needs of people caring for someone with a rare dementia, improving their access to specialised resources and support. By extending iSupport to this population, it contributes to advancing dementia care inclusivity and broadening the understanding of rare dementias. A feasibility study is underway to assess iSupport RDCs acceptability, with prospects for cultural adaptations to benefit carers globally.

## 1. Introduction

This study focuses on the adaptation of iSupport, an online intervention by the World Health Organization (WHO) aimed at supporting carers of people with dementia. iSupport was initially developed for those caring for individuals with Alzheimer’s disease (AD) and vascular dementia. However, the prevalence of over 250 dementia types, including rare types, presents unique challenges for carers due to different presentations and ages of onset [1].

It is estimated that there are 944,000 people living with dementia in the UK [2]. Alzheimer’s disease (AD) accounts for around 50–75% of diagnoses and vascular dementia for around 20% [2]. AD and vascular dementia typically occur in people over the age of 65 [1]. Consequently, most services focus on supporting people with these diagnoses and their carers, who are typically older adults [3]. However, rare forms of dementia make up between 5 and 15% of the people diagnosed with dementia in the UK [1,4]. Rare dementias are more common in people under the age of 65, and it has been estimated that there are 44,000 people living with a young-onset dementia in the UK [5]. Although more common in people under the age of 65, rare dementias can also occur in older adults [6].

People living with young-onset dementia may be at different life stages than those over the age of 65; they may still be of working age or have young children [7]. Partners who are caring for someone with a young-onset dementia may have to manage working, caring for their partner with dementia, and ageing parents or young children [3].

Rare dementias often present with symptoms like visual difficulties, behavioural changes, or language problems [1], which are different from the memory problems associated with more common dementias like AD and vascular dementia [8]. Due to these differences, people might be referred to services that might struggle to recognise these symptoms as indicative of dementia and are often inappropriate and ill equipped to offer support to people with rare dementia and their carers [4,8]. Consequently, carers can experience heightened emotional strain and a sense of isolation due to the lack of tailored support [9,10]. The objective of this study was to create a flexible intervention that can better support the specific challenges encountered while caring for people with rare dementias. The aim is to ensure carers receive more effective and personalised support to improve their wellbeing.

Research highlights the importance of customised interventions for carers, particularly in addressing the challenges posed by rare dementia types [9,10]. The option of using the internet to deliver supportive interventions offers a practical and cheap option to carers who may not have the time to travel to in-person sessions [9,11,12,13,14]. iSupport for dementia carers, initially designed for prevalent dementia types, offers a potential option [11]. It provides information on dementia, relaxation strategies for the carer, and practical caring advice. This study adapted iSupport in collaboration with informal carers and healthcare professionals to meet the needs of carers of people with rare dementias.

**Figure 1:**
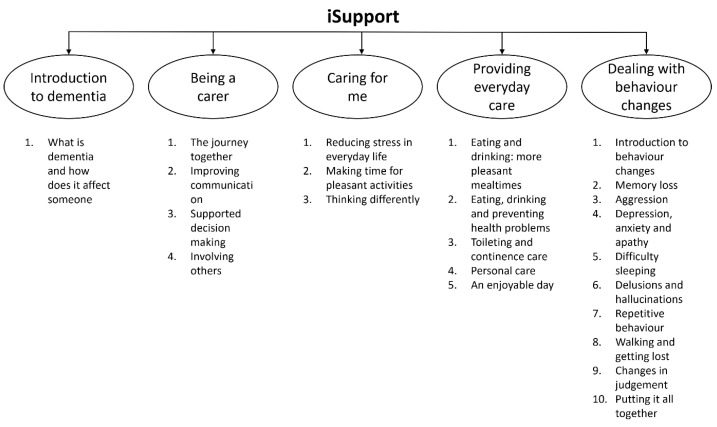
The five modules of iSupport.

iSupport is divided into five modules, as outlined below (Figure 1). Information is provided on a topic, and then carers are given the opportunity to reflect on the information and apply it to their own situations by engaging in interactive exercises. “Check your understanding” boxes give a scenario description and then ask what the appropriate response to the situation would be. Short pieces of practical advice are also presented in “tip” boxes to highlight the point and catch the attention of the carers.

iSupport adaptations are undertaken through a licence agreement with the WHO, and an adaptation guide is provided, aiding in the translation and customisation of the programme for various languages and cultures [15]. The licence allows modification of the iSupport resource, provided the researchers have adhered to the WHO adaptation guidelines [15] and can demonstrate evidence to support the modifications, and the WHO must approve them before they are implemented. The licence for this adaptation was held by the Dementia Services Development Centre (DSDC) at Bangor University. In this study, the aim was to adapt iSupport for the specific needs and preferences of carers of people with rare dementias.

## 2. Materials and Methods

This study used the Standards for Reporting Qualitative Research (SRQR) to provide transparent and full descriptions of this research procedure (Supplementary Table S1). These standards were selected due to their flexible application to different qualitative methods [16].

**Figure 2:**
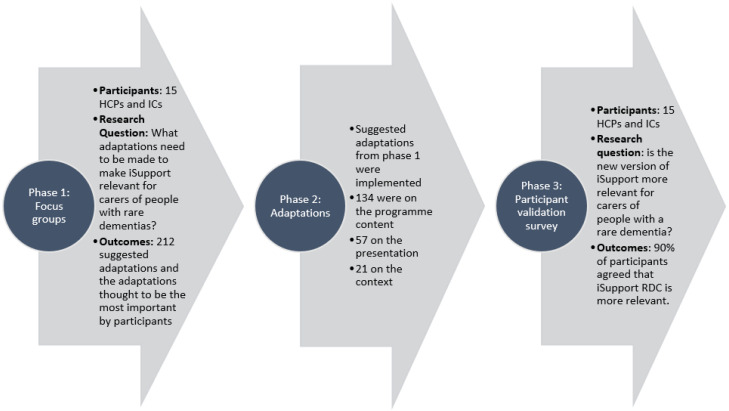
The adaptation process.

The adaptation was created in three phases (Figure 2): (1) focus groups and interviews were undertaken that generated and prioritised topics for adaptation; (2) analysis of ideas and identifying areas for adaptation; and (3) a follow-up survey to ascertain whether the adaptations made based on phases 1 and 2 were acceptable to the participants. The adapted version of iSupport RDC was then forwarded to the WHO for final approval ahead of further research and implementation. Minor changes were suggested by the WHO, and approval was granted.

## 3. Phase 1—Focus Groups

To make changes to iSupport, the adaptation guide [15] describes several requirements.

a. Adaptions and potential changes will be generated by focus groups. There should be two focus groups of six to eight informal carers and six to eight healthcare professionals. The participants’ characteristics should be varied to ensure representation; for our study, we focused on different genders, types of dementia, ethnicity, and relationships with the person with dementia.
b. The adaptation guide suggests two steps for the focus groups:
  (1) Participants should go through iSupport individually and make note of any changes they would like to see.
  (2) Focus groups should be arranged to discuss these changes in depth with the participants.

Using participant feedback to make decisions about adaptations should require rigorous methods, such as consensus methods. Consensus methods are decision-making tools that are often used in healthcare research, where stakeholders discuss and achieve agreement on a particular topic [17,18].

After exploring various consensus methods like Delphi, consensus development panels, and the nominal grouping technique (NGT) [17], NGT emerged as the best choice for the focus groups. Unlike the Delphi method, which involves lengthy survey rounds and consensus panels used in conferences [17], NGT offers a one-session approach, allowing equal participation and benefiting those uncomfortable in group settings or with limited availability [18,19]. NGT is a group-based method to achieve consensus by investigating stakeholders’ perspectives [19]. It consists of a structured group interaction [18], and results are achieved in one session. Participants can interact with each other within the structure of the sessions, offering all participants an equal opportunity to express their ideas, which is beneficial for participants who are less comfortable in group settings. This method was also beneficial to the participants, who were busy carers and healthcare professionals, since they only needed to attend a one-off session. Consequently, the NGT was selected to be used in the focus groups.

The five steps of NGT were followed in this study [18]:

(1) Introduction: The facilitator explains the topic, how the focus group will run, and what is expected of participants. They introduce this research question.
(2) Silent idea generation: The participants are given time to think and write down their responses to this research question. This is carried silently so that the responses are not biassed in any way.
(3) Idea sharing: The facilitator asks each participant for one of their ideas until all ideas are written on a shared whiteboard.
(4) Clarification of ideas: The facilitator asks participants to share more details of their ideas to clarify their meaning.
(5) Prioritisation of ideas: Participants rank the ideas that they think are the most important. This is carried out anonymously to avoid response bias.

### 3.1. Recruitment

Participants, both Informal Carers (IC) and Health Care Professionals (HCP), were recruited from the Rare Dementia Support (RDS) network as a part of the larger RDS Impact study [4] and through the authors professional contacts with HCPs. RDS is a University College London (UCL)-led service that provides support to people living with seven types of rare dementia and those around them [1]. The types of rare dementia supported are posterior cortical atrophy (PCA), primary progressive aphasia (PPA), frontotemporal dementia (FTD), and Lewy body dementia (LBD). As part of opting-in to become a member of the RDS network, ICs and HCPs have the opportunity to be contacted about participating in research.

The following eligibility criteria were used for both IC and HCP participants: participants must have the capacity to consent to participate in this study; they must be over 18 years old; and they must have experience working with or caring for people living with PCA, PPA, LBD, and FTD. These dementia types were selected because they do not initially present with memory problems.

Participants were excluded from this study if they were unable to understand written English, since it was necessary for participants to share their views on the English version of iSupport or did not have access to a computer or tablet with an internet connection.

A short advertisement describing the project was circulated via email to potential participants in the RDS network and to professional contacts. Once a participant expressed an interest in taking part in the iSupport adaptation study, the primary author (BNM) sent the participant information sheet and arranged an online meeting using the videoconferencing platform Go To Meetings (GTM) to discuss eligibility and outline this study procedure.

### 3.2. Consent Procedures

Ethical approval was obtained from research ethics committees at UCL as a part of a programme titled the Rare Dementia Support Impact project: The impact of multicomponent support groups for those living with rare dementias [4] (ref. 8545/004) and Bangor University (ref. 2020-17057). The consent procedures were the same for both ICs and HCPs and followed the procedure set out in the RDS Impact project protocol [4]. This states that an audio-visual recording of the consenting process takes place via GTM, whereby the researcher read the consent form to the participant and video-recorded their stated verbal consent to each point within the form.

## 4. Phase 1—Procedure

Following informed consent, participants received an online or printed PDF version of the English translation of iSupport at least two weeks before taking part in the NGT, as recommended by the WHO adaptation guide [15].

As suggested in the WHO adaptation guide, participants were asked the following questions:

- What is your general impression of iSupport?
- Are there any suggestions to make it more relevant to carers of people with rare dementias?

Participants were asked to write down their answers to these questions and make general notes on the iSupport materials and content before the focus group sessions were arranged. Participants were then contacted via email or using an online poll to arrange suitable dates for the focus group.

Work and caring commitments for both HCPs and ICs made scheduling focus groups difficult. To offer a more convenient option and reduce the number of people unable to attend, individual interviews were also offered [20]. Dates for focus groups were suggested, and if participants were unavailable, individual interviews were offered, or participants could provide written feedback. This resulted in five individual interviews and three focus group sessions. Three participants opted to provide written feedback only (Table 1). The procedure for the individual interviews followed the same NGT structure as the focus groups.

In addition to the WHOs predefined questions, each focus group and interview concluded with a request for participants to identify the five adaptations they thought were the most important to make iSupport relevant for carers of people with rare dementias. This process, termed ‘prioritised changes’ in NGT and in this paper, involved participants selecting and ranking adaptations they believed were essential. The offer of interviews instead of focus groups was a modification, as NGT is a group consensus method. The addition of interviews meant that the clarification step of the NGT was not conducted with all participants present. In a traditional NGT focus group, they would be able to vote on the suggestions from all participants; however, in this modified NGT, it was only the suggestions from either focus group participants or their own suggestions. Consequently, a consensus was not reached on which changes could be made from the focus groups and interviews alone. Therefore, based on previous work by Bergerød et al. [19], phase three was included to seek feedback on the preliminary suggestions.

### 4.1. Data Analysis

The online focus groups and interviews were recorded on GTM and transcribed verbatim by the primary author (BNM). Some participants provided feedback via email or added comments directly to the PDF version of iSupport. The data analysis were conducted in two steps: (1) thematic analysis of interview and focus group transcripts and written feedback; and (2) comparison of the prioritised changes between the two groups.

#### 4.1.1. Thematic Analysis

To analyse adaptation suggestions, the primary author followed the six-step process outlined by Braun and Clarke [21] for thematic analysis. The generation of themes involved:

Familiarisation: Immersing in the data to gain an understanding of the content, identifying patterns, and noting initial ideas for potential themes.

Coding: Systematically coding the data and identifying meaningful sections relevant to adaptation suggestions.

Generating Themes: Grouping together similar codes to form preliminary themes that capture underlying concepts or ideas related to adaptation suggestions.

Reviewing Themes: Including the second and third authors (GW and CL) to assess the consistency of the preliminary themes against the coded data, refining or combining themes where necessary.

Defining Themes: Clearly defining and naming final themes and ensuring they accurately represent the data.

Writing a Report: Documenting the findings, including the identified themes and their interpretations, for reporting purposes.

#### 4.1.2. Researchers’ Characteristics and Reflexivity

All authors have experience working with or caring for people living with dementia in both clinical and research settings. All authors have backgrounds in psychology; the primary author (BNM), responsible for the data collection, analysis, and write-up, have academic experience in psychology and clinical experience working as a care assistant in a care setting for people with dementia. The second author (GW), responsible for validating the data analysis and editing the manuscript, has academic experience in psychology and dementia research. The third author (CL), responsible for validating the data analysis and editing the manuscript, worked as a clinical psychologist with carers and people with dementia and has undertaken dementia research. The fourth author (EB), responsible for editing the manuscript, has academic experience in psychology and dementia research, and the fifth author (SC), also responsible for editing the manuscript, is a neuropsychologist and has experience in dementia research.

#### 4.1.3. Comparison of Prioritised Changes

The identified prioritised changes that both groups of participants (ICs and HCPs) selected and ranked were aligned under the themes generated in the thematic analysis. The alignment was completed by the primary author (BNM) and then discussed with co-authors for validation (GW and CL).

The overlap of prioritised changes between the two groups was compared by calculating the percentage of the themes that were present in both groups, weighted by the number of votes for each. This involved assessing the level of agreement with the prioritised changes. The weighted percentage enabled a comparison of the priorities for each group.

## 5. Phase 1—Results

### 5.1. Participant Information

Participants were six ICs (4 F) and nine HPCs (9 F), which was in line with the number of participants recommended by McMillan [18], as well as with the WHO adaptation guidelines that recommended six to eight people in each group [15]. One of the HCPs was known to the primary author (BNM), and two others were recruited through professional contacts of the third author (CL). Approximately 7 of the 15 participants (47%) provided their ages: four HCPs (*M* = 39) and three ICs (*M* = 71) (Table 2). The remaining eight declined to provide their ages.

### 5.2. Thematic Analysis

The suggested adaptations were analysed thematically, and three levels of themes were identified (Table 3). One-hundred and thirty-four of the suggested adaptations relate to the programme content, 57 to the presentation, and 21 to the context.

#### 5.2.1. Theme 1: Presentation

The theme of the presentation included feedback on how the iSupport intervention was visually and structurally perceived by participants. Within the subtheme of design, the focus group discussions highlighted the significance of using images in a more “age-appropriate”-IC4 style to resonate with more users. Participants emphasised the need for better organisation within the intervention, suggesting improvements in the navigation and structure of the content. Concerns were raised about the website design, prompting suggestions for enhanced usability and accessibility features. Additionally, feedback was given on the overall volume of information, which was described as “overwhelming”-IC6, indicating the necessity for adaptations that balance comprehensiveness without overwhelming users. The language subtheme also fell under the presentation theme of iSupport. Participants found elements of iSupport to be patronising, such as the “check your understanding” exercises and the “well done!” at the end of each lesson. There were also phrases that were described as outdated and not person-centred, such as "saying “Making time for yourself WILL make you feel better”. Participants felt that it was important to say that things MIGHT make you feel better but that the tips in iSupport will not work for everyone. Highlighting the need to be aware of the language used in interventions for carers. The main message from the suggested adaptations on presentation was that carers are often short on time and stressed, so they need an intervention that is easy-to-use, not overwhelming, and does not patronise them.

#### 5.2.2. Theme 2: Content

Content emerged as the theme that captured the largest number of suggestions regarding the amount of information and psychological support offered within the intervention. Under the subtheme of a rare or younger focus, participants stressed the importance of including information about the symptoms of rare dementia and tailoring content to meet the needs of younger individuals affected by dementia. Examples that were focused on rare dementias and their symptoms were suggested, along with insights into the specific challenges associated with rare dementias. The subtheme of psychological help/wellbeing included the need for self-care prioritisation, seeking professional advice, and more robust psychological support tailored for People with Dementia (PwD). Normalising emotions and promoting enjoyable activities were identified as essential components of effective support. The findings from the focus groups also included suggestions for signposting participants to other services for support, information, and healthcare. This was to avoid adding more information to an already overwhelming resource, to advise carers to seek professional advice in certain circumstances, and to offer more specific information for different diagnoses, which people can choose to look into depending on its relevance to their situation. The final subtheme was practical care advice, in which participants suggested tailoring for rare dementias and the specific symptoms that may occur, including more on communicating with people who have aphasia and that in FTD, incontinence may be a behavioural rather than physical symptom. They also mentioned the potentially faster progression of rare dementias, which means that advanced planning is more important in the early stages of dementia and that adapting care as the dementia progresses is an important thing for iSupport to mention. Lastly, participants indicated a need for more legal advice on employment, driving (for the early stages of dementia), and deprivation of liberty laws (for the later stages).

#### 5.2.3. Theme 3: Context

The context theme included feedback on the situational and individual perspectives that can influence the relevance of iSupport. Under the subtheme of person/relationship-centred care, participants highlighted the importance of individual experiences, emphasising that not all tips or strategies would universally apply. Acknowledgment was given to the uniqueness of each carer, PwD, and situation, and suggestions were made that the intervention needed to respect and accommodate these differences. Participants emphasised the necessity of approaching care and support with a person-centred view, recognising the importance of tailored strategies for varying relationships and contexts.

These three level 1 themes and level 2 themes demonstrate the complex nature of the feedback provided by informal carers and healthcare professionals. The depth of discussions around presentation, content, and context highlights the diverse needs when caring for people with rare dementias. Addressing these different perspectives and recommendations is crucial for enhancing the relevance and effectiveness of the iSupport intervention.

### 5.3. Comparison of Priortised Changes

The overlap of suggestions between the two groups was compared by calculating the percentage of suggestions that were present in both groups, weighted by the number of votes for each idea (Table 4). There was 74% (*n* = 56) agreement on the five most important adaptations between the ICs and HCPs. Twenty suggestions (26%) were not mentioned in both groups, so it was assumed to show disagreement in the priorities of the groups. One participant endorsed only two suggestions, and another only three.

## 6. Phase 2—Adaptations

The original proposal was to use the prioritised changes to inform the adaptations to iSupport based on the endorsement and consensus around the most important ideas from the ICs and HCPs. However, most of the adaptations were small and easy to implement, so this research team (BNM, GW, and CL) reviewed the data and discussed which of the suggested adaptations could be implemented. Decisions on which adaptations to make were based on how often they were suggested, whether they were practical to implement (time- or cost-effective, generalisable), and whether they were in line with the principles of the original intervention [22]. The RDS Impact team of researchers and the WHO reviewed the adaptations to ensure the additional information provided was factually correct.

There were 212 suggested adaptations that this research team reviewed, of which 194 (92%) were practical, generalisable, and kept to the principles of iSupport. Examples of adaptations that were not made included the removal of entire modules and replacing the word ‘carer’ with ‘journey partner’ since the word carer is widely used and understood. The implementation of the suggested adaptations and subsequent edits was completed by the primary author in consultation with this research team, RDS healthcare professionals, research professionals, and informal carers. The RDS Impact team of researchers and the WHO reviewed the adaptations to ensure the additional information provided was factually correct.

The suggested adaptations from the focus groups, interviews, and written feedback were grouped into the three themes derived from the thematic analysis: presentation, content, and context.

### 6.1. Theme Level 1 a: Presentation

#### 6.1.1. Design

The feedback on the design of iSupport was mixed. Participants’ comments on the design include:

‘*The drawings are childish. It undermines my role as a carer and as an adult carer.*’—IC4

‘*Playful images trivialise the important content.*’—IC6

**Figure 3:**
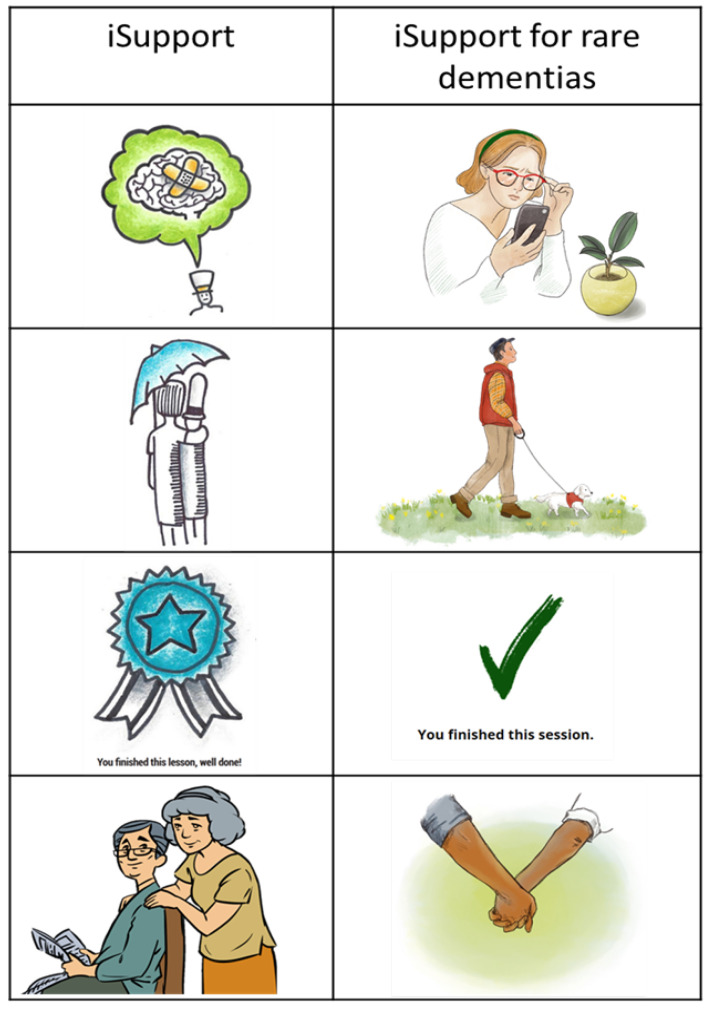
iSupport images before and after adaptation.

An illustrator was commissioned to create new, age-appropriate images (Figure 3). The image on the front cover was changed since participants pointed out that an older couple might not be representative of people with rare dementias. They suggested that the cover should be something that can apply to anyone regardless of their age, gender, and ethnicity.

In general, the participants liked the organisation of the modules and lessons. The tips were highlighted as being particularly useful. However, the Check Your Understanding exercises were described as patronising, and having incorrect responses could be:

‘*unhelpful for carers who are already worried about doing things wrong.’*—HCP2

**Figure 4:**
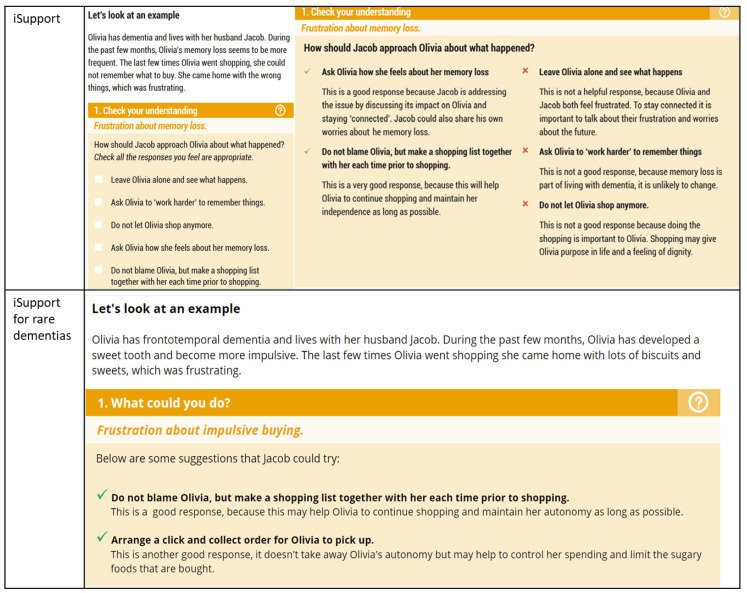
Check your understanding exercises before and after adaptation.

Instead, it was suggested that lists of positive responses to try were included for each example scenario, with the clarification that they might not work for everyone (Figure 4).

Participants giving feedback on the PDF version of iSupport suggested that the:

‘*size is overwhelming for busy carers*’—IC6

However, they appreciated that when accessing iSupport online, carers could read the sections that were relevant to them and skip the rest.

#### 6.1.2. Language

The language of iSupport was described as patronising by most of the participants. In particular, at the end of each lesson, where it says, ‘You’ve finished this lesson, well done!’, consequently, the ‘well done!’ was removed. The phrase ‘pleasant activities’ was also highlighted as patronising, and participants suggested replacing it with ‘enjoyable activities’.

A few terms were highlighted as being out-dated. For example, the term ‘wandering’ is no longer used, so this was removed. ‘Pick’s disease’ is not in the DSM V [23] and was identified by HCPs as out-dated. However, the term was used by ICs in an RDS support group that was attended by the primary author (BNM), so both terms were included with the following amendment: ‘frontotemporal dementia, previously known as Pick’s disease’.

### 6.2. Theme Level 1 b: Content

Most of the suggested changes (134) were to the content. Considering the different symptoms and age groups affected by rare dementias, it made sense that the content was the area that needed adapting the most. Examples of these adaptations include making the module on symptoms and behaviour changes more relevant by adding information on visual changes, movement problems, and apathy.

#### 6.2.1. Rare Dementia Focus

The participants discussed the need to change the focus of iSupport to younger people who may need specific advice on employment, driving, and talking to children about dementia. They also suggested that since younger people are typically more active, the suggested enjoyable activities in Module 3 needed to reflect that. Another suggestion was the need to change the symptoms in Module 5, since rare dementias present differently.

‘*Section on behaviours—need specific, in-depth information about sleep and hallucinations, disinhibition and fTD specific behaviours, and perception (specific to each type of dementia).*’—HCP3

‘*More examples of younger people living with FTD.*’—IC1

**Figure 5:**
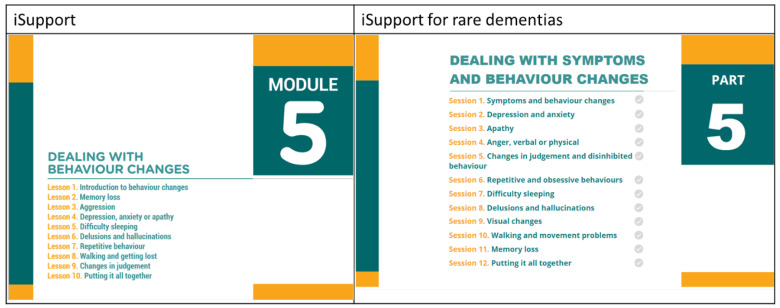
Module/Part five content before and after adaptation.

In response to this feedback, sections on employment, driving, and talking to children were added, and the symptoms were amended to include visual changes, changes to mobility, and more detail on apathy (Figure 5).

#### 6.2.2. Psychological Help

Another point raised was that there should be more content that focuses on psychological help and wellbeing for both the person with dementia and the carer so links to websites offering counselling for people living with dementia and their carers were added.

#### 6.2.3. Signposting

It was suggested that links to support services and where to find additional information for each topic would be useful. Links to websites of reputable organisations were added so that people could find information on specific topics that are relevant for their situation without increasing the size of iSupport.

#### 6.2.4. Practical Care Advice

While there was some information in iSupport on the importance of planning ahead to when the person with dementia can no longer make their own decisions, participants said that they would like further information beyond the standard iSupport provision. As a result, the section on advice about Powers of Attorney and wills was expanded. Another practical care adaptation was that participants requested more emphasis on the importance of adapting care as dementia progresses or even at different times of the day. This was added in the format of tip boxes, explaining that certain responses to situations will work sometimes but not others and that when dealing with a degenerative condition, adaptability is important.

### 6.3. Theme Level 1 c: Context

#### Person/Relationship-Centred Care

Participants’ feedback suggested that the advice in iSupport needed to be more context-specific and person-centred. It should be made clear that the advice would not work for everyone, and what might work sometimes might not work at other times.

‘*You should say that exercises may not work for everyone.*’—HCP6

Another suggestion was to respect the person with dementia’s preferences prior to their diagnosis, for example, by maintaining activities that they enjoyed previously and the style of clothing that they liked to wear.

Another context-specific point that was raised by the participants was the different relationships with the person with dementia. Since rare dementias can affect younger people, their carers may be caring for a parent, spouse, or child with a rare dementia. The examples were edited to include relationships with a younger focus, for example, fewer examples involving grandparents with dementia and more parents or adult children with dementia. Another suggestion was to add same-sex couples to the examples. A spreadsheet was created to ensure the examples used throughout the iSupport for rare dementia included the different types of rare dementia and the sex of the carers. A random name generator was also used to select different names in the clients’ vignettes to reflect the diverse UK population after participants pointed out the lack of cultural diversity in the names used in the examples (Supplementary Table S2).

## 7. Phase 3—Procedure

A phase of work was added to explore whether the adaptations made were useful. Participants were emailed the adapted version of iSupport and were invited to give their opinion on the changes they previously suggested in the focus groups, interviews, and written feedback. The changes were summarised into simple, focused statements based on the seven themes (Table 3) [24]. The participants were asked to rate each statement (Table 5) using a 5-point Likert scale, from strongly agree to strongly disagree. A five-point scale was chosen since a number between four and seven has been suggested to be optimal, with five or seven being the most used [25]. Descriptive statistics of the survey data were calculated, and additional suggested changes from participants were discussed by this research team as above.

## 8. Phase 3—Results

**Figure 6:**
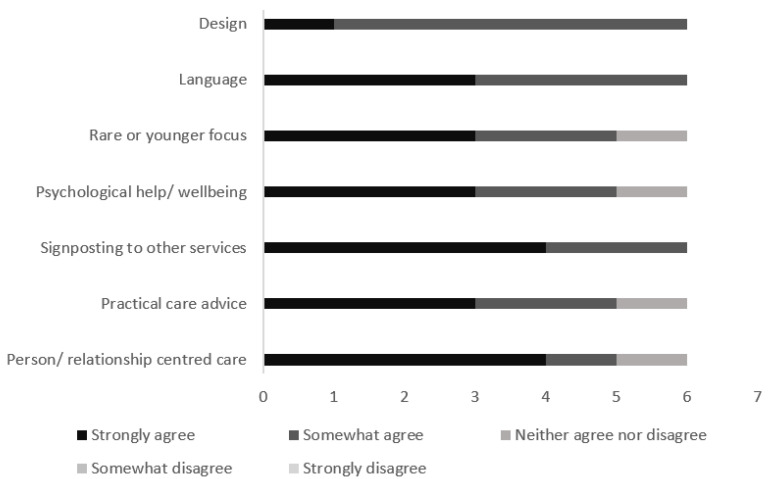
Responses to the participant validation survey questions based on the seven themes.

A total of 6 of the 15 participants (40%) responded to the survey. Half of the responses (50%) strongly agreed that the adapted version of iSupport was improved in the seven areas; 40% agreed, and 10% neither agreed nor disagreed (Figure 6). None of the participants responded with disagree or strongly disagree. One participant further suggested adding information about applying for a disabled parking badge and mentioned swaying as a symptom of LBD. This was discussed with the research team, who decided to add these changes.

## 9. Discussion

This study adapted iSupport, an e-health intervention developed by the World Health Organisation, for carers of people with rare dementia. The adaptations reflect the lived experiences of both family and professional carers, ensuring their expertise was embedded in the process. Thus, a new resource was created, tailored to the unique needs of these carers, to help support their wellbeing, knowledge, and skills. The priorities of the two groups based on which adaptations were most important were compared. HCPs and ICs agreed on 74% of the most important adaptations, suggesting that both groups wanted similar things from the intervention. While there was a high level of agreement, there were differences in the priorities of HCPs and Ics. The variations in priorities between HCPs and Ics likely stem from their distinct vantage points. Ics, driven by their intimate caregiving experiences, prioritise adaptations that address emotional and practical challenges encountered daily [26]. In contrast, HCPs, guided by clinical expertise, may emphasise evidence-based practises or structured interventions [26]. Further qualitative exploration could investigate motivations, bridge the gap between clinical objectivity and the emotional aspects of caregiving, and enhance intervention tailoring.

### 9.1. Integration with Previous Work, Implications, and Transferability

The themes of presentation, content, and context were in line with the Ecological Validity Model (EVM) of adapting interventions [27]. This model suggests that when adapting interventions for different cultural settings, the language, people, metaphors, and content need to be considered. Although this was not a cultural adaptation, the participants’ feedback overlapped with these ideas.

There was also considerable overlap with participants’ feedback from other iSupport adaptations [28]. The UK adaptation for young carers found that participants also thought the language in iSupport was patronising in places, that the right or wrong answers in the exercises were inappropriate for carers who may worry about being judged, and that the examples in the studies needed to be changed to a more familiar family situation.

iSupport was developed to be easily translated into other languages and for use in lower- to middle-income countries [11]. This is further supported by the adaptations in India, Brazil, and Portugal that highlight the importance of using simple language to reflect the reading levels in these countries [29,30,31,32]. The fact that participants in the young carer adaptation [28] and this study perceived some of the languages in iSupport as patronising could be due to different reading levels, as suggested by Baruah et al. [30], Oliveira et al. [31], and Teles et al. [29], but could also be explained by cultural differences in interpretation. Consequently, alternative but simple language was used in the adaptations to counter these perceptions. 

Another change that had been identified in several adaptations, including this one, was that the “correct” and “incorrect” responses for the “check your understanding” exercises were not person-centred. There is not always a “correct” response when caring for someone with dementia, and what works for some people may not work for others [28,29].

### 9.2. Strengths and Limitations

The focus group and interviews were conducted in April 2022, when the COVID-19 restrictions were easing in the UK. Since the participants were carers and healthcare professionals who had close contact with clinically vulnerable populations and the restrictions were unpredictable, the data collection was completed online using video-conferencing software. As well as avoiding any risk of spreading COVID-19, this also meant that participants did not have to travel to take part in this study. This resulted in the recruitment of participants from different locations in the UK. It also meant that carers did not have to organise alternative care arrangements to take part in the focus groups, and healthcare professionals did not have to take time off work to travel. Despite this, finding suitable timeslots for a focus group proved difficult, illustrating the demands that both HCPs and ICs had on their time. The adaptation of the NGT methods to offer interviews or focus groups offered more flexibility, which is more person-centered, and enabled more participants to take part. However, this meant that participants being interviewed could only vote for their own ideas, potentially limiting the prioritised changes. It could, however, decrease the risk of bias from peer pressure, conformity, or an over-reliance on more confident group members to voice their opinions [20].

Participants with the four types of rare dementia were invited to take part; however, no carers of people with PCA responded, so the advice for carers of PCA was based on the suggestions from HCPs and researchers. Using focus group methods to include the perspectives of both healthcare professionals and informal carers improved the validity of the adaptation.

The demographic data were limited by the number of responses to the questionnaire; however, the sample consisted of mainly British (80%) and female participants (87%). The mainly female sample might have biassed the feedback; however, it may also be representative of the population; 11.3% of women in the UK are carers compared to 8.6% of males [33]. Traditional roles may be important in this context; women may be socialised to be more nurturing, whereas men may have difficulty with the emotional aspects of caring for a family member [34]. The age range was large (32–78 years old), and aside from a lack of carers for people with PCA, the type of dementia was varied. The majority of the carers were spouses. Future testing of iSupport RDC should focus on recruiting carers of people with PCA, male carers, a more ethnically diverse sample with different relationships to the PwD, in order to test its applicability to different carer subgroups. The all-female composition of the data collection and analysis team (BNM, GW, and CL) may have influenced participants’ responses and interpretations. Nonetheless, conflicting research exists on whether interviewer gender impacts disclosed information [35].

While the number of participants was aligned with WHO guidelines and the NGT methodology [15,18], it is possible that not all adaptations were proposed. The concept of theoretical saturation guided data collection (the point at which no new information is being found from focus groups or interviews), and the purpose of NGT is to collect ideas until there are no new ones (the data saturation point has been reached), but a specific saturation point was not defined before the focus groups [17,18,36]. Theoretical saturation is a term from grounded theory [37] and is used to determine how many data collection sessions are needed. The nominal grouping method that was used in this study involved asking the participants for their ideas until there were no new suggestions made, and therefore it was assumed that the data saturation point had been reached. Using data from semi-structured interviews, researchers found that 93% of the total themes were identified in the first six interviews and that this increased to 97% after 12 interviews [38]. Guidelines on reaching data saturation suggest that six interviews are sufficient to achieve 80% data saturation and that 12 interviews would increase it to 90% [38].

The response rates for the demographics and feedback questionnaires were 40% and 53%, respectively. This is within the acceptable response rates suggested by Kerlinger (1973, as cited in [39]). However, with a small sample size to begin with, higher responses would have been preferred. Previous studies using similar methods also found low response rates to the participant feedback step [19]. This could have been due to time constraints for busy ICs and HCPs or questionnaire fatigue [40]. To improve the response rate, the demographic data could have been collected as interview questions during the consent meeting. Recognising the limitations of response rates, future studies could benefit from using different data collection techniques tailored to participants’ preferences and time constraints. Offering varied data collection modes, integrating incentives, refining questionnaire design for clarity and relevance, and initiating personalised outreach strategies could improve participation. Additionally, employing a phased approach to data collection, allowing for adjustments based on real-time feedback, might optimise overall engagement.

Digital exclusion concerns in online interventions must also be addressed, with approximately 7–8% of UK adults lacking reliable internet access [41,42,43]. To mitigate this, the generic iSupport version is available as a downloadable PDF, ensuring accessibility for those without internet access. Participants in this study were given the option to view the PDF of iSupport or request a paper copy, as well as the option to attend telephone interviews if there were any issues with an internet connection. The rare dementia adaptation will also be provided in PDF format, addressing potential digital exclusion concerns.

## 10. Conclusions

iSupport is an eHealth intervention that has global reach but is targeted at older people with memory-led dementias. This adaptation has been created using co-design methods to involve people caring for someone with a rare dementia as well as healthcare professionals and researchers to further increase the reach of iSupport to an alternative carer population.

Currently underway is a pilot study to assess the acceptability of iSupport RDC. Following this, iSupport will be freely available, and service providers can feel confident that this evidence-informed product can be suggested as part of care and support packages. It will be accessible on the Bangor University website and through the WHO website, both online and downloadable as a PDF. In the future, it could be culturally adapted for carers in other countries to further increase its reach.

Drawing from this study, recommendations for service providers, researchers, and policymakers could involve fostering collaborations between stakeholders, emphasising the importance of tailored interventions, and advocating for the integration of iSupport RDC into existing care frameworks. Additionally, ongoing research and continual feedback loops should inform improvements to ensure its sustained relevance in meeting the needs of carers of people with rare dementias.

## Figures and Tables

**Table 1:** Number of ICs and HCPs that attended interviews, focus groups, or provided written feedback.

Feedback Delivery Method	Number of Participants
Focus group discussion 1	2 HCPs
Focus group discussion 2	3 HCPs
Focus group discussion 3	2 ICs
Individual interviews	2 HCPs, 3 ICs
Written Feedback Only	1 IC, 2 HCPs

Note: Healthcare professionals (HCPs) and informal carers (ICs).

**Table 2:** Participant demographics.

IC Demographics (*n* = 6)		
Gender	Female	4 (67%)
Relationship to PwD	Spouse	5 (83%)
	Child	1 (17%)
Type of dementia	LBD FTD Familial FTD	3 (50%) 1 (17%) 1 (17%)
	PPA	1 (17%)
Ethnicity *	English/Welsh/Scottish/Northern Irish/British	4 (67%)
	Other: Mediterranean	1 (17%)
HCP demographics (*n* = 9)		
Gender	Female	9 (100%)
Ethnicity *	English/Welsh/Scottish/Northern Irish/British	8 (89%)

Note: * One IC and one HCP declined to provide ethnicity data.

**Table 3:** Three levels of themes were identified from the participants’ feedback.

Theme Level 1	Theme Level 2	Subthemes
a. Presentation	1. Design	Images Organisation Website design Overall volume
	2. Language	Patronising Out-dated Not person-centred
b. Content	3. Rare or younger focus	Rare dementia symptoms Focus on younger people Examples Challenges of rare dementias
	4. Psychological help/wellbeing	Prioritise yourself Seek professional advice Psychological help for PwD Emotions are normal Enjoyable activities
	5. Signposting for other services	For information For support For healthcare
	6. Practical care advice	Practical care advice Importance of adapting care Planning Dealing with symptoms Legal
c. Context	7. Person/ relationship-centred care	Tips will not work for everyone All experiences are different Respect the individual Different relationships

**Table 4:** Prioritised changes per theme and the number of times they were endorsed.

Level 2 Themes	ICs	HCPs
Design	9	5
Language	6	3
Rare or younger focus	7	16
Psychological help/wellbeing	3	2
Signpost to other services	3	4
Practical care advice	4	1
Person/relationship-centred care	3	10

**Table 5:** Participant survey and corresponding themes.

Level 2 Theme	Participant Survey
Design	The new images are more appropriate for carers of people with rare dementias.
2. Language	The changes to the language have made the new version more appropriate for carers of people with rare dementias.
3. Rare/younger focus	The new version is focused more on rare dementias and younger people.
4. Psychological help/wellbeing	The new information on psychological wellbeing and where to find help is useful for carers of people with rare dementias.
5. Signposting to other services	The added links for more information are useful for carers of people with rare dementias.
6. Practical care advice	The practical advice in the new version is more appropriate for those caring for someone with a rare dementia.
7. Person/relationship-centred care	The new version is more focused on caring for the individual and makes it clear that not all advice will work for everyone.

## Data Availability

The participants of this study did not give written consent for their data to be shared publicly, so due to the sensitive nature of this research, supporting data are not available.

## Supplementary Materials

The following supporting information can be downloaded at: https://www.mdpi.com/article/10.3390/ijerph21010047/s1; Supplementary Table S1: Standards for reporting qualitative research; and Supplementary Table S2: Case study diversity.

## References

[1] RDS Rare Dementia Support [RDS}. Published 2022. https://www.raredementiasupport.org/.

[2] Alzheimer’s Society Alzheimer’s Society/Demographics. Published 2023. https://www.alzheimers.org.uk/.

[3] Gibson A.K., Anderson K.A., Acocks S. (2014). Exploring the Service and Support Needs of Families with Early-Onset Alzheimer’s Disease. Am. J. Alzheimer’s Dis. Other Dement..

[4] Brotherhood E.V., Stott J., Windle G., Barker S., Culley S., Harding E., Camic P.M., Caufield M., Ezeofor V., Hoare Z. (2020). Protocol for the Rare Dementia Support Impact study: RDS Impact. Int. J. Geriatr. Psychiatry.

[5] Hall M., Sikes P. (2020). ‘It’s just limboland’: Parental dementia and young people’s life courses. Sociol. Rev..

[6] Harvey R.J., Roques P., Fox N.C., Rossor M.N. (1996). Non-Alzheimer dementias in young patients. Br. J. Psychiatry.

[7] Mayrhofer A.M., Mathie E., McKeown J., Goodman C., Irvine L., Hall N., Walker M. (2020). Young onset dementia: Public involvement in co-designing community-based support. Dementia.

[8] Suárez-González A., Harding E., Zimmerman N., Hoare Z., Brotherhood E., Crutch S. (2020). The impact of the first UK Covid-19 lockdown on carers and people living with low prevalence dementia: Results from the Rare Dementia Support survey. medRxiv.

[9] Naunton Morgan B., Windle G., Sharp R., Lamers C. (2022). eHealth and Web-Based Interventions for Informal Carers of People with Dementia in the Community: Umbrella Review. J. Med. Internet Res..

[10] Sullivan M.P., Williams V., Grillo A., McKee-Jackson R., Camic P.M., Windle G., Stott J., Brotherhood E., Crutch S.J., on behalf of the Rare Dementia Support (RDS) Research Team (2022). Peer support for people living with rare or young onset dementia: An integrative review. Dementia.

[11] Pot A.M., Gallagher-Thompson D., Xiao L.D., Willemse B.M., Rosier I., Mehta K.M., Zandi D., Dua T., iSupport Development Team (2019). iSupport: A WHO global online intervention for informal caregivers of people with dementia. World Psychiatry.

[12] Rathnayake S., Moyle W., Jones C., Calleja P. (2019). mHealth applications as an educational and supportive resource for family carers of people with dementia: An integrative review. Dementia.

[13] Chaudhry B.M., Smith J. (2021). RefineMind: A Mobile App for People with Dementia and Their Caregivers. The Next Wave of Sociotechnical Design.

[14] Mahmood A., Kim H., Chang C.F., Kedia S., Arshad H., Dillon P.J. (2023). mHealth Apps Use and Their Associations with Healthcare Decision-Making and Health Communication Among Informal Caregivers: Evidence from the National Cancer Institute’s Health Information National Trends Survey. Am. J. Health Promot..

[15] WHO (2017). ISupport Version 1.0. Adaptation and Implementation Guide (Unpublished).

[16] O’Brien B.C., Harris I.B., Beckman T.J., Reed D.A., Cook D.A. (2014). Standards for reporting qualitative research: A synthesis of recommendations. Acad. Med..

[17] Waggoner J., Carline J.D., Durning S.J. (2016). Is there a consensus on consensus methodology? Descriptions and recommendations for future consensus research. Acad. Med..

[18] McMillan S.S., King M., Tully M.P. (2016). How to use the nominal group and Delphi techniques. Int. J. Clin. Pharm..

[19] Bergerød I.J., Braut G.S., Fagerdal B., Gilje B., Wiig S. (2021). Developing a Next-of-Kin Involvement Guide in Cancer Care-Results from a Consensus Process. Cancer Nurs..

[20] Lambert S.D., Loiselle C.G. (2008). Combining individual interviews and focus groups to enhance data richness. J. Adv. Nurs..

[21] Braun V., Clarke V. (2006). Using thematic analysis in psychology. Qual. Res. Psychol..

[22] Yardley L., Morrison L., Bradbury K., Muller I. (2015). The Person-Based Approach to Intervention Development: Application to Digital Health-Related Behavior Change Interventions. J. Med. Internet Res..

[23] American Psychiatric Association (2013). Diagnostic and Statistical Manual of Mental Disorders.

[24] Williams A. (2003). How to … Write and Analyse a Questionnaire. J. Orthod..

[25] Asún R.A., Rdz-Navarro K., Alvarado J.M. (2016). Developing Multidimensional Likert Scales Using Item Factor Analysis: The Case of Four-point Items. Sociol. Methods Res..

[26] Robinson A., Eccleston C., Annear M., Elliott K.-E., Andrews S., Stirling C., Ashby M., Donohue C., Banks S., Toye C. (2014). Who knows, who cares? Dementia knowledge among nurses, care workers, and family members of people living with dementia. J. Palliat. Care.

[27] Sit H.F., Ling R., Lam A.I.F., Chen W., Latkin C.A., Hall B.J. (2020). The Cultural Adaptation of Step-by-Step: An Intervention to Address Depression Among Chinese Young Adults. Front. Psychiatry.

[28] Masterson-Algar P., Egan K., Flynn G., Hughes G., Spector A., Stott J., Windle G. (2022). iSupport for Young Carers: An Adaptation of an e-Health Intervention for Young Dementia Carers. Int. J. Environ. Res. Public Health.

[29] Teles S., Napolskij M.S., Paúl C., Ferreira A., Seeher K. (2021). Training and support for caregivers of people with dementia: The process of culturally adapting the World Health Organization iSupport programme to Portugal. Dementia.

[30] Baruah U., Loganathan S., Shivakumar P., Pot A.M., Mehta K.M., Gallagher-Thompson D., Dua T., Varghese M. (2021). Adaptation of an online training and support program for caregivers of people with dementia to Indian cultural setting. Asian J. Psychiatry.

[31] Oliveira D., Jacinto A.F., Gratao A.C.M., Ottaviani A.C., Ferreira C.R., Monteiro D.Q., Barham E.J., Orlandi F.d.S., de Cruz K.C.T., Correa L. (2020). Translation and cultural adaptation of iSupport in Brazil. Alzheimer’s Dement..

[32] Xiao L.D., McKechnie S., Jeffers L., De Bellis A., Beattie E., Low L.-F., Draper B., Messent P., Pot A.M. (2021). Stakeholders’ perspectives on adapting the World Health Organization iSupport for Dementia in Australia. Dementia.

[33] Dahlberg L., Demack S., Bambra C. (2007). Age and gender of informal carers: A population-based study in the UK. Health Soc. Care Community.

[34] Sharma N., Chakrabarti S., Grover Nidhi Sharma S., Grover S. (2016). Gender differences in caregiving among family-caregivers of people with mental illnesses. World J. Psychiatry.

[35] Miyazaki A.D., Taylor K.A. (2008). Researcher interaction biases and business ethics research: Respondent reactions to researcher characteristics. J. Bus. Ethics.

[36] Carlsen B., Glenton C. (2011). What about N? A methodological study of sample-size reporting in focus group studies. BMC Med. Res. Methodol..

[37] Glaser B.G., Strauss A.L., Strutzel E. (1968). The Discovery of Grounded Theory; Strategies for Qualitative Research. Nurs. Res..

[38] Guest G., Namey E., Chen M. (2020). A simple method to assess and report thematic saturation in qualitative research. PLoS ONE.

[39] Rabin L., Barr W., Burton L. (2005). Assessment practices of clinical neuropsychologists in the United States and Canada: A survey of INS, NAN, and APA Division 40 members. Arch. Clin. Neuropsychol..

[40] Egan K.J., Pinto-Bruno C., Bighelli I., Berg-Weger M., van Straten A., Albanese E., Pot A.-M. (2018). Online Training and Support Programs Designed to Improve Mental Health and Reduce Burden Among Caregivers of People with Dementia: A Systematic Review. J. Am. Med. Dir. Assoc..

[41] Helsper E.J., Reisdorf B.C. (2017). The emergence of a “digital underclass” in Great Britain and Sweden: Changing reasons for digital exclusion. New Media Soc..

[42] Litchfield I., Shukla D., Greenfield S. (2021). Impact of COVID-19 on the digital divide: A rapid review. BMJ Open.

[43] Prescott C. (2021). Internet Users, UK 2020.

