# Supplementary material for: Adaptation of an eHealth Intervention: iSupport for Carers of People with Rare Dementias
Source: Int J Environ Res Public Health. 2023 Dec 28;21(1):47. doi: 10.3390/ijerph21010047 (PMC10815602; doi:10.3390/ijerph21010047)
Supplement: Supplementary file 1 [file ijerph-21-00047-s001.zip › Supplementary Table S1. Standards for Reporting Qualitative Research.pdf]

Standards for Reporting Qualitative Research (SRQR) Table

SRQR Headings	Description	Page Numbers
Title	Concise description of the study topic identifying the study as qualitative, indicating approach, or data collection methods	Title…
Abstract	Summary of key elements of the study	Abstract: Page 1
Problem formulation	Description and significance of the problem; review of relevant theory and empirical work; problem statement	Introduction: Pages 4-6
Purpose or research question	Purpose of the study and specific objectives or questions	Introduction: Pages 4-6
Qualitative approach and research paradigm	Qualitative approach, guiding theory, and rationale	Methods: Pages 6-8
Researcher characteristics and reflexivity	Researchers' characteristics that may influence the research	Data analysis: Page 13
Context	Setting and salient contextual factors	Discussion: Pages 24-28
Sampling strategy	How and why participants were selected; criteria for deciding when no further sampling was necessary	Recruitment: Pages 9-10<br>Discussion: Page 27
Ethical issues pertaining to human subjects	Documentation of approval by an appropriate ethics review board and participant consent	Ethics & consent procedures: Pages 9-10
Data collection methods	Types of data collected and details of data collection procedures	Phase 1- Methods: Pages 10-13<br>Phase 3-Methods: Pages 22-23

Data collection instruments and technologies	Description of instruments and devices used for data collection	Phase 1- Focus groups: Pages 7-9<br>Phase 3-Methods: Pages 22-23
Data processing	Methods for processing data before and during analysis	Data analysis: Page 12
Data analysis	Process by which themes were identified and the researchers involved in data analysis	Data analysis: Pages 12-13
Techniques to enhance trustworthiness	Techniques to enhance trustworthiness and credibility of data analysis	Data analysis: Pages 12-13
Units of study	Number and relevant characteristics of participants and level of participation (can be reported in results)	Phase 1- Results: Page 14<br>Phase 3- Results: Page 24
Synthesis and interpretation	Main findings, which may include development of a theory or model	Phase 1- Results: Page 14<br>Phase 2- Adaptations: Pages 17-22<br>Phase 3- Results: Page 24
Links to empirical data	Evidence to substantiate findings (e.g., quotes)	Phase 2- Adaptations: Pages 17-22
Integration with prior work, implications, transferability	Summary of findings, explanation of how findings connect to conclusions of earlier scholarship, discussion of scope of application, and unique contribution to field	Integration with previous work, implications, and transferability: Pages 25-26
Limitations	Trustworthiness and limitations of findings	Strengths and Limitations: Pages 26-27
Conflicts of interest	Potential sources of influence on study conduct and conclusions	Conflicts of interest: Page 28
Funding	Sources of funding and other support	Funding: Page 28
